# TRANSNATIONAL PARENT-CARE AND FILIAL PIETY: THE PARADOX OF IDEALIZED TRADITIONAL IMMIGRANT VALUES

**DOI:** 10.1093/geroni/igae098.3759

**Published:** 2024-12-31

**Authors:** Joonsik Yoon

**Affiliations:** Syracuse University, Syracuse, New York, United States

## Abstract

For families from highly filial societies, such as those from East Asian countries, providing care to parents in later life has long been considered an important value and duty. However, providing parent-care to older adults in modern societies can present many practical challenges for families. When adult children emigrate abroad while the parents stay, the physical distance separating the transnational families presents additional difficulties in meeting filial expectations and providing practical care. In this qualitative ethnographic study, ten first-generation Korean Americans were interviewed in depth using an open-ended, semi-structured protocol. These interviews focused on respondents’ experiences and concerns surrounding transnational parent-caregiving for parents living back in South Korea. A grounded theory approach was used to adapt the research focus to accommodate emerging thematic elements as the interviews progressed. Most respondents expressed a strong preference for idealized and traditional models of filial piety. They not only felt a strong obligation to support and care for their parents as they aged, but many even stated that they wanted to co-reside with their parents. This is notable since the practice is not only outdated in the West, but it has rapidly fallen out of favor and use in Korea. This preference for seemingly outdated traditional filial values stood in stark contrast to the respondents’ acknowledgement that there were significant obstacles in achieving their preferred care goals. I conclude with a discussion of the respondents’ preferences for seemingly outdated and unachievable filial ideals.

